# Randomness and Irreversiblity in Quantum Mechanics: A Worked Example for a Statistical Theory

**DOI:** 10.3390/e23121643

**Published:** 2021-12-07

**Authors:** Yves Pomeau, Martine Le Berre

**Affiliations:** Laboratoire d’Hydrodynamique, Ladhyx, CNRS UMR 7646, Ecole Polytechnique, 91128 Palaiseau, France; yves.pomeau@gmail.com

**Keywords:** quantum jumps, irreversibility, fluorescence, Kolmogorov-like model, Everett’s interpretation

## Abstract

The randomness of some irreversible quantum phenomena is a central question because irreversible phenomena break quantum coherence and thus yield an irreversible loss of information. The case of quantum jumps observed in the fluorescence of a single two-level atom illuminated by a quasi-resonant laser beam is a worked example where statistical interpretations of quantum mechanics still meet some difficulties because the basic equations are fully deterministic and unitary. In such a problem with two different time scales, the atom makes coherent optical Rabi oscillations between the two states, interrupted by random emissions (quasi-instantaneous) of photons where coherence is lost. To describe this system, we already proposed a novel approach, which is completed here. It amounts to putting a probability on the density matrix of the atom and deducing a general “kinetic Kolmogorov-like” equation for the evolution of the probability. In the simple case considered here, the probability only depends on a single variable θ describing the state of the atom, and p(θ,t) yields the statistical properties of the atom under the joint effects of coherent pumping and random emission of photons. We emphasize that p(θ,t) allows the description of all possible histories of the atom, as in Everett’s many-worlds interpretation of quantum mechanics. This yields solvable equations in the two-level atom case.

## 1. Introduction

The transition from Newtonian mechanics to quantum mechanics in the early years of the twentieth century has been a major step in the progress of our understanding of the world. This transition was more than a change in equations because it also involved a deep change in our understanding of the limits of human knowledge. It included, from the very beginning, a statistical interpretation of the theory. In other words, quantum mechanics is not fully predictive and cannot be so. The introduction of statistical methods to describe nature was not new, of course. Statistical concepts were introduced in physics to interpret classical (non-quantum) laws as a way to describe complex systems with many degrees of freedom, such as assemblies of many atoms in a macroscopic volume of fluid. The mathematical theory behind the statistical approach in classical physics is ergodic theory because no human being has enough computational power to solve Newton’s equations [1] with the initial data (position and velocity) of too many particles. Nowadays, one cannot solve the classical equations of motion of more than a few thousand particles. In classical mechanics, a slightly more subtle point makes it difficult to predict the future from the initial data in the long run. This is related to the ergodic (Ergodic is the term used by Kolmogorov, although the common word is now chaotic or Anosovian if the trajectories are Lyapunov unstable [2]) properties of classical dynamics: a flow is ergodic, chaotic, or Anosovian if a small disturbance or inaccuracy in the initial conditions is amplified in the course of time. This property of ergodicity is very hard to prove for given systems. As far we are aware, this has been proven to be true [3] only for systems of hard spheres making elastic collisions, and the proof is highly non-trivial. In the two examples (many particles and/or ergodicity of classical dynamics), the statistical method of analysis is just a way to describe systems given the imperfect knowledge of the initial conditions and their overwhelming abundance. On the contrary, quantum mechanics needs, from the very beginning, a statistical interpretation, a point that has raised controversies. To many, it seemed strange to postulate (see later for the precise meaning of this word in this context) a statistical interpretation of a theory that looks to be “deterministic” in the sense that the dynamical equations (Schrödinger or Dirac equations, including the interaction with the electromagnetic (EM) field) look well posed with a unique solution for given initial data. What is called “determinism” is, however, not as well defined as one could believe at first. There is a clearly defined *mathematical* meaning of the concept based on the notion (seemingly first understood by Newton) that, for given initial data (position and velocities of particles moving in vacuo), there is a well-defined future for a dynamical system obeying differential equations of a finite order in time. A superficial view could be that because the equations of non-relativistic quantum mechanics are mathematically “deterministic” and of the first order in time, a complete understanding of the initial data is enough to predict the future. The fallacy of this concept is in the word “complete”. Because measurements of the initial conditions are made with quantum devices, there is a *fundamental* uncertainty in the initial conditions due to the limited accuracy of those measurements, a point made by Heisenberg [4]. This is central to our discussion: In the case of the emission of photons by an atom in an excited state, the instant of the emission cannot be predicted from measurements of the initial state of the atom. This fundamental question of the determination of the time of decay of an atom by emission of a photon was answered by Dirac [5] in a masterpiece of science in the context of black-body radiation, which is different from the one devoted to quantum jump statistics for a two-level atom pumped by a laser field treated here.

Note that the word “quantum jump”, which is currently used for a single atom that emits photons when submitted to an EM field, may be ambiguous, particularly because the interaction between the atom and the emitted photon has a typical intrinsic time and period of the EM wave involved; therefore, it does not make sense to make statements for times shorter than this “intrinsic” time scale. The wave function of the full system—atom plus photons outside—changes continuously in time because when an atom initially in the excited state emits a photon, the resulting state is made of the atom in its ground state, plus an outgoing photon added to the EM field, and the amplitude of this new state (emitted photon, EM field, plus atom in the ground state) grows continuously from zero. When the initial state of the atom is a superposition of the ground state and of the excited state, it may go through the excited state under the effect of the Rabi oscillations and can then jump back to the ground state by emitting a photon, or the atomic state may follow Rabi oscillations without emitting any photons, with the atomic state evolving as a superposition of the ground and excited states until the next emission of a photon (which could occur only when the atomic state goes through the excited state). In summary, both possibilities (emission of a photon or no emission) exist in different universes in the Everett sense, as explained below.

After the early days of this grand history of the birth of quantum mechanics, a somewhat arcane field of knowledge had to ask whether such a theory with seemingly well-posed dynamical equations (Schrödinger and Dirac equations) has a kind of fundamental statistical interpretation. This is the aim of the present paper, which focuses on a worked problem, the fluorescence of a single atom. In the list of obscure concepts introduced to make the quantum description match the real data, let us quote what is often called the “reduction (or collapse) of the wave packet (or wave function)”. Our aim is not to decide on the measure problem in quantum mechanics, which was the object of many debates and is still a controversial topic. However, let us note that the difficulties related to the conservation of the total probability are removed in Everett’s theory.

In 1957, Everett introduced [6] a convincing explanation compatible both the idea of reduction of the wave packet and the constraint of unitarity of the evolution, or of conservation of the probability in the statistical interpretation. Everett’s idea is that each outcome of a measurement creates a new universe with a subsequent history consistent with the result of this measurement but disconnected from other universes corresponding each to another outcome of the measurement. This profound idea makes everything consistent at the price of introducing a direction of time. This direction of time plays the same role as the one introduced to explain the arrow of time of thermodynamics; namely, it represents the physical impossibility of reversing the history of a peculiar system. Said otherwise, the statistics introduced by quantum mechanics are there, in principle, to make averages over all universes corresponding to various outcomes of a measurement. As said above, because we are discussing something related to physics and not philosophy, there are consequences of this line of reasoning in the physical and mathematical picture of processes. This relies on definite equations for probability distributions, of which we shall give an example below.

In the case of the fluorescent light emitted by a single atom, the characteristic time associated with a quantum jump is very short, of the order of the laser period [5] and much smaller than the Rabi period. This property allows us to make the Markov approximation leading to our Kolmogorov-like equation for the evolution of the probability distribution of a single variable θ describing the trajectory of the atom.

In Section 2, we present our model equation for the evolution of the single parameter θ controlling the atomic state and derive the statistics of the emission times $t_{i}$ with and without the pump field. In Section 3, we explain why Everett’s theory is useful in interpreting our statistical description of the fluorescence of a single atom.

## 2. A Model Physical Problem

The spontaneous emission of photons by an assembly of atoms in thermal equilibrium was considered by Einstein [7] and by Dirac [5] as fundamentally random. Einstein used statistics to describe an atom interacting with black-body radiation. In this case, there is a continuous process of excitation of the ground state by the black-body radiation, but practically, this is not a very efficient process compared to the excitation by a resonant monochromatic beam, which we shall consider. Thanks to the progress of experimental atomic physics, in 1986, Hans Dehmelt [8,9,10] observed the leaping of electrons from one atomic state to another in individual atoms. This sudden transition of a tiny object (such as an electron, ion, molecule, or atom) from one of its discrete energy states to another has been called a quantum jump since Niels Bohr, who put this concept forward for discontinuous events, although Schrödinger (and others) strongly objected to their existence, postulating instead that they are not instantaneous.

Here, we study a simpler case, the emission of light by a two-level atom, an interesting worked example from the point of view of the statistical interpretation of quantum mechanics.

### 2.1. Towards a Full Statistical Theory of the Emission Process

We shall outline the principles of a statistical treatment that is able to describe both the emission of photons and the optical Rabi oscillations in the case of a single pumped two-level atom, detailed in [11]; then, we shall explain how to derive the probability distribution of the time intervals between two successive photon-emission events. This was based upon the property that, in such an interval, the atom does make unhindered Rabi oscillations, and that the emission of a photon is a phenomenon seen as instantaneous. This is, of course, one basic feature of a Markov process because we consider quick jumps occurring at random with a probability depending on the state of the system and, possibly, on the absolute time. For such a phenomenon, the Kolmogorov equation seems to be the right tool to describe the state of an atom because this kind of equation describes the evolution of the probability distribution of a system under the effects of two processes, one leading to a deterministic dynamics, the other to random quasi-instantaneous events, as just written. Let Θ(t) be the set of time-dependent parameters changing with time, with the time derivative $\partial_{t}\Theta =v\left(\Theta \right)$, a function of Θ. In the deterministic phase, the conservative and normalized probability p(Θ,t) obeys the equation

(1) $$\partial_{t}p\left(\Theta ,t\right)+\partial_{\Theta }\left(v\left(\Theta \right)p\left(\Theta ,t\right)\right)=0,$$

where $\partial_{t}$ is here and elsewhere for the derivative with respect to time, and $\partial_{\Theta }$ for the derivative with respect to Θ (This is actually a gradient in general because Θ has more than one component, but this only complicates the writing in an unessential way).

Kolmogorov equations add a right-hand side representing instantaneous transitions (or jumps) occurring at random instants of time to this equation, represented by a positive-valued function $\Gamma (\Theta^{\prime }|\Theta )$. During a small interval of time dt, if the system is in state Θ, it quickly jumps to state $\Theta^{\prime }$ with probability $\Gamma (\Theta^{\prime }|\Theta )dt$ so that the Kolmogorov equation describes both the deterministic dynamics and the jump process and reads [12]:

(2) $$\partial_{t}p\left(\Theta ,t\right)+\partial_{\theta }\left(v\left(\Theta \right)p\left(\Theta ,t\right)\right)=\int d\Theta_{1}\Gamma \left(\Theta |\Theta_{1}\right)p\left(\Theta_{1},t\right)-p\left(\Theta ,t\right)\int d\Theta^{\prime }\Gamma \left(\Theta^{\prime }|\Theta \right).$$

In the right-hand side, the first positive term describes the increase in probability of the Θ-state due to jumps from other states to Θ. The second term represents the loss of probability because of jumps from Θ to any other state $\Theta^{\prime }$. By integration over Θ, one finds that the $L^{1}$-norm ∫dΘp(Θ,t) is constant (if it converges, as we assume).

Let us now consider a two-level atom whose wave function is of the form

(3) $$\Psi_{at}\left(t\right)=\left(\cos\left(\theta \left(t\right)\right)|g>+ie^{i\omega t}\sin\left(\theta \left(t\right)\right)|e>\right)e^{i\varphi },$$

where $\dot{\theta }$, the time derivative of θ(t), is equal to Ω/2 between two jumps, Ω being the Rabi frequency. The Kolmogorov equation deals explicitly with the probability distribution p(θ,t) for the atomic state, here indexed by a single variable θ.

In the right-hand side of Equation (2), the probability $\Gamma (\theta ;\theta^{\prime })$ for the atom to make a quantum jump from the state θ towards the state $\theta^{\prime }$ is proportional to $\delta (\sin\theta^{\prime })$ (where δ(.) is the Dirac distribution) because any jump lands on $\theta^{\prime }=0$ in the interval [−π/2,π/2], and this probability is proportional to $\gamma \sin^{2}\theta$ because it comes from the state $a_{1}$ with the squared amplitude $\sin^{2}\theta$, and γ is the emission rate of the atom in the excited state calculated by Dirac [5]. Therefore,

(4) $$\Gamma \left(\theta |\theta^{\prime }\right)=\gamma \;\sin^{2}\theta \;\delta \left(\sin\theta^{\prime }\right).$$

Thus, the Kolmogorov equation for the two-level atom illuminated by a resonant pump field is

(5) $$\frac{\partial p}{\partial t}+\frac{\Omega }{2}\frac{\partial p}{\partial \theta }=\gamma \left(\delta \left(\sin\theta \right)\int_{-\pi /2}^{\pi /2}d\theta^{\prime }\;p\left(\theta^{\prime },t\right)\sin^{2}\theta^{\prime }-p\left(\theta ,t\right)\sin^{2}\theta \right),$$

Introducing a probability distribution depending on a continuous variable, θ here, which amounts to putting a probability on the elements of the atomic density matrix, is a way to take into account all possible trajectories emanating from the emission of a single photon, with a new value of the number of photons radiated in any direction at each quantum jump. Average values of a time-dependent quantity that depends on θ can be calculated via the probability distribution p(θ,t), which is a π-periodic function with a finite jump at θ=0, but smooth elsewhere. This procedure allows us to deal correctly with the infinite number of possible trajectories, since Boltzmann’s genius lies precisely in transforming the classical statistical theory based on unknown initial conditions into statistics for an ensemble of indeterminate trajectories.

We insist that our description of the fluorescence of a single two-level atom goes beyond solving Heisenberg equations (which is impossible anyway without making a strong hypothesis because of the infinite number of degrees of freedom of the EM field). Here, as in the quantum mechanical frame, the infinite number of degrees of freedom are taken care of because they represent the fast phenomena, which are well approximated in Dirac’s calculation of the coefficient γ for the black-body radiation calculus. Moreover, the whole story before and after each rapid event is told here through the balance terms written in the right-hand side of the Kolmogorov equation, which has a built-in conservation law of the total probability at any time, a serious advantage with respect to the quantum treatments using a Lindblad equation [13], which is difficult to handle [14].

Because it is linear, Equation (5) can be solved in a Laplace transform, but the general solution in time requires the inversion of a Laplace transform, which can be done only formally. There are two constraints: (i) The probability p(θ,t) is positive or zero and (ii) the total probability $\int_{-\pi /2}^{\pi /2}d\theta \;p\left(\theta ,t\right)$ is unity at any time, which reflects the unitary evolution of the atomic state (the integral of the square modulus of the wave function is constant and equal to one). It is relatively easy to check that they are fulfilled, since $\int_{-\pi /2}^{\pi /2}d\theta \;p\left(\theta ,t\right)$ is constant and p(θ,t)≥0 at any positive time if p(θ,0)≥0. Solutions in various limits are derived in [11]. The factors $\sin^{2}\theta$ on the right-hand side are there to take into account that a quantum jump occurs only if the atom is in the excited state, which has probability $\sin^{2}\theta$. The negative term on the right-hand side is the loss term representing the decrease in the amplitude of the excited state by jumps to the ground state, whereas the positive one is for the increase in the amplitude of the ground state when a jump takes place.

The populations of the two levels, or probabilities for the atom to be in the excited or in the ground state at time *t*, are, respectively,

(6) $$\rho_{1}\left(t\right)=\int_{-\pi /2}^{\pi /2}d\theta^{\prime }\;p\left(\theta^{\prime },t\right)\sin^{2}\theta^{\prime }.$$

and

(7) $$\rho_{0}\left(t\right)=\int_{-\pi /2}^{\pi /2}d\theta^{\prime }\;p\left(\theta^{\prime },t\right)\cos^{2}\theta^{\prime }.$$

Their sum is one, as it should be, if p(θ,t) is normalized to one.

From (5), one can derive an equation for the time derivative of $\rho_{1}\left(t\right)$ and $\rho_{0}\left(t\right)$ by multiplying (5) by $\sin^{2}\theta$ and by $\cos^{2}\theta$ and integrating the result over θ. This gives

(8) $${\dot{\rho }}_{1}=-\frac{\Omega }{2}\int_{-\pi /2}^{\pi /2}d\theta^{\prime }\;\sin^{2}\theta^{\prime }\frac{\partial p}{\partial \theta }-\gamma \left(\int_{-\pi /2}^{\pi /2}d\theta^{\prime }\;p\left(\theta^{\prime },t\right)\sin^{4}\theta^{\prime }\right),$$

and

(9) $${\dot{\rho }}_{0}=-\frac{\Omega }{2}\int_{-\pi /2}^{\pi /2}d\theta^{\prime }\;\cos^{2}\theta^{\prime }\frac{\partial p}{\partial \theta }+\gamma \left(\int_{-\pi /2}^{\pi /2}d\theta^{\prime }\;p\left(\theta^{\prime },t\right)\sin^{4}\theta^{\prime }\right).$$

On the r.h.s of the rate Equations (8) and (9), the first term, proportional to the Rabi frequency Ω, describes the effect of the Rabi oscillations, whereas the second term, proportional to γ, displays the effect of the quantum jumps responsible for the photo-emission. Because p(θ,t) includes both the fluctuations due to the quantum jumps and the streaming term, the right-hand side of (8) and (9) represents the new history beginning at each step. After integration by parts, (8) and (9) become

(10) $${\dot{\rho }}_{1}\left(t\right)=-{\dot{\rho }}_{0}\left(t\right)=\int_{-\pi /2}^{\pi /2}d\theta \;p\left(\theta ,t\right)\;\left(\frac{\Omega }{2}\sin2\theta -\gamma \sin^{4}\theta \right).$$

Note that the set of Equations (8) and (9), or (10), is not closed. It cannot be mapped into equations for $\rho_{1}\left(t\right)$ and $\rho_{0}\left(t\right)$ only because their right-hand sides depend on higher momenta of the probability distribution p(θ,t), momenta that cannot be derived from the knowledge of $\rho_{1}\left(t\right)$ and $\rho_{0}\left(t\right)$. The unclosed form of (8) and (9) is a rather common situation. To name a few cases, the BBGKY hierarchy of non-equilibrium statistical physics makes an infinite set of coupled equations for the distribution functions of systems of interacting (classical) particles [15], where the evolution of the one-body distribution depends explicitly on the two-body distribution, which depends itself on the three-body distribution, etc. In the theory of fully developed turbulence, for instance, the average value of the velocity depends on the average value of the two-point correlation of the velocity fluctuations, depending itself on the three-point correlations, etc. Fortunately, one can solve the Kolmogorov Equation (5) via an implicit integral equation [11]; then, there is generally no need to manipulate an infinite hierarchy of equations as in those examples.

In the present case, one can say, following Everett, that the probability distribution p(θ,t) allows one to make averages over the states of the atom in different universes, each being labeled by a value of θ at a given time *t*. As written above, physical phenomena such as the observation of a quantum state decay measured by emission of a photon are relative to the measurement apparatus that takes place in the universe associated with the observer. At every emission of a photon, a new history begins, represented by the right-hand side of (9). In summary, the creation of new universes at each step defines a Markov process, which can be described by a Kolmogorov statistical picture, and cannot be considered as a deterministic process depending in a simple way on averaged quantities, such as population values.

### 2.2. Quantum Jump Statistics

To illustrate how one can use the Kolmogorov equation, we derive the time-dependent probability of photo-emission by a single atom, first without any pump field, then in the presence of a resonant laser.

We consider first an isolated atom initially in pure state $\Psi_{at}\left(0\right)$ given by (3) with $\theta \left(0\right)=\theta_{0}$. The solution of (5) with Ω=0 (no pump) and $p\left(\theta ,0\right)=\delta \left(\theta -\theta_{0}\right)$ is

(11) $$p\left(\theta ,t\right)=\left(1-q\left(t\right)\right)\delta \left(\theta \right)+q\left(t\right)\delta \left(\theta -\theta_{0}\right)\;\mathrm{with}\;q\left(t\right)=e^{-(\gamma \sin^{2}\theta_{0})t}.$$

The evolution of the probability that the atom is in the excited state at time *t* is given by (6), and the emission of a photon occurs randomly in time with a rate:

(12) $$\dot{\rho_{1}}=-\gamma \sin^{2}\theta \left(t\right)\rho_{1}\left(t\right).$$

Once the atom “jumps” to its ground state, it cannot emit another photon; then, the emission of a photon, if recorded, is a way to measure the state of the atom. The solution of (12) leads to the population of the excited state

(13) $$\rho_{1}\left(t\right)=\sin^{2}\theta_{0}e^{-(\gamma \sin^{2}\theta_{0})t}$$

when taking into account the initial condition, and the photo-emission rate is

(14) $$\dot{\rho_{1}}\left(t\right)=-\gamma \sin^{4}\theta_{0}e^{-(\gamma \sin^{2}\theta_{0})t}.$$

The probability of photo-emission in the interval (0,∞) is the integral of $\dot{\rho_{1}}$:

(15) $$\int_{0}^{\infty }\gamma \sin^{4}\theta_{0}\;e^{-(\gamma \sin^{2}\theta_{0})t}dt=\sin^{2}\theta_{0},$$

which means that the final state of the coupled system of the atom plus the emitted photon field is

(16) $$\Psi \left(\infty \right)=\sin\theta_{0}\left|g,1>+e^{i\phi^{\prime }}\cos\theta_{0}\right|g,0>$$

where the indices (1, 0) correspond to the one and zero photon states, respectively. The relation (15) means that if we consider *N* atoms initially prepared in a given pure state with $\theta \left(0\right)=\theta_{0}$, namely, with total energy $N\sin^{2}\theta_{0}\;\hbar \omega$, we get, at infinite time, *N* atoms in the ground state and $N\sin^{2}\theta_{0}$ photons of individual energy ℏω. In the final state, only a fraction of them, $N\sin^{2}\theta_{0}$, jump from the excited state to the ground state with the emission of a photon; the others, $N\cos^{2}\theta_{0}$, simply stay in the ground state [16].

In the case of an atom submitted to a resonant pump field, the atom will emit photons at random times, forming a point process. Here, we assume that the process is Markovian, but more generally, any process with time-dependent history is completely characterized by its conditional intensity function $\lambda (t|H_{t})$, the density of points at time *t*, where $H_{t}$ is the history of the emission activity up to time *t*, and the time interval probability distribution is given by the relation $\ell \left(\tau \right)=\lambda \left(\tau |H_{\tau }\right)e^{-\int_{0}^{\tau }\lambda \left(t|H_{t}\right)dt}$. In the present Markovian case, the conditional intensity of the point process, which is the probability of emission of a photon at time *t*, only depends on the value of θ at this time; therefore, one simply has $\ell \left(\tau \right)=\lambda \left(\tau \right)e^{-\int_{0}^{\tau }\lambda \left(t\right)dt}$. From (8), we deduce

(17) $$\lambda \left(t\right)=\gamma \sin^{4}\theta \left(t\right).$$

**Figure 1:**
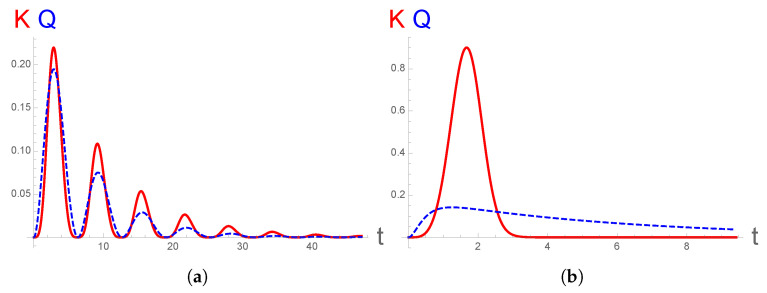
Inter-emission time distribution ℓ(t) in two opposite cases: (**a**) for weak and (**b**) for strong dissipative rates (with the respect to the Rabi frequency). The solid red curves are for our Kolmogorov statistical theory (Equation (18)). The dashed blue curves display the delay function deduced in [18,19] for the same values of Ω/γ, which are equal to 3.33 in (**a**) and 1/6 in (**b**).

In this relation, the exponent 4 comes from two conditions: One in which the atom is in the excited state, and the other in which it emits a photon, as in (14), which describes an emission without any pump field. With a pump field, in between two successive emission times, the atom undergoes Rabi oscillations with θ(t)=Ωt/2, assuming that a photon is emitted at time t=0. Therefore, the inter-emission time distribution for an atom driven by a resonant pump is given by the expression [17]:

(18) $$\ell \left(\tau \right)=\gamma \sin^{4}\left(\frac{\Omega }{2}\tau \right)\;\;e^{-\gamma \int_{0}^{\tau }\sin^{4}\left(\frac{\Omega }{2}t\right)},$$

which gives $\int_{0}^{\infty }\ell (\tau )d\tau =1$, as expected. The result is shown in Figure 1 in the two opposite limits of large and small values of the ratio Ω/γ and is compared to the delay function derived in [18,19] (which does not have the standard form expected for a Markovian process). For the case of a strong input field, Ω>γ, the two methods approximately agree; see Figure 1a. However, they differ noticeably in the opposite case, which is shown in Figure 1b. For weak laser intensity (or strong damping), the Kolmogorov derivation gives a mean delay between successive photons of order $\tau_{K}={\left(\Omega^{4}\gamma \right)}^{-1/5}$, which decreases slowly as the damping rate γ increases, which seems reasonable. In the same limit, the dressed atom method leads to $\tau_{Q}=\gamma /\Omega^{2}$, a time scale much longer than the inverse of γ, and increasing with the damping rate, a result that seems to go against intuition [20].

### 2.3. Relationship with Planck-Einstein Theory

The above analysis of the spontaneous emission was devoted to an atom (or an ensemble of independent atoms) initially prepared in the pure state

(19) $$\Psi_{at}\left(t\right)=\cos\left(\theta_{0}\right)\left|g>+i\sin\left(\theta_{0}\right)\right|e>.$$

In this case, the non-diagonal component of the atomic density matrix evolves as

(20) $$\rho_{01}\left(t\right)=i\sin\left(\theta_{0}\right)\cos\left(\theta_{0}\right)e^{-\gamma \sin^{2}\theta_{0}\;t}.$$

This case—the so-called “coherent case”—displays a rate of emission of photons that is not equal to γ, the line-width of the excited state, but is equal to $\gamma \sin^{2}\theta_{0}$. Then, $\dot{\rho_{1}}\left(t\right)$ depends in a non-trivial way on the atomic state. This points to a potentially interesting feature because the rate decreases when θ decreases; therefore, the atom is maintained in the excited state longer than in the case of black-body radiation, where the decay rate is γ, as was deduced by Planck and Einstein. In the latter case, the atoms are in thermal equilibrium (an incoherent state with $\rho_{10}=0$), with a probability (1−p) of being in the ground state or *p* of being in the excited state. At equilibrium, the probability $p_{eq}\left(\theta \right)$ is

(21) $$p_{eq}\left(\theta ,0\right)=\left(1-p\right)\delta \left(\theta \right)+p\delta \left(\theta -\pi /2\right).$$

Taking expression (21) as an initial condition, the problem reduces to the one treated in Section 2.2 with Ω=0 and $\theta_{0}=\pi /2$. The solution of the Kolmogorov equation is then given by (11), and the non-diagonal component of the atomic density matrix is given by (20). The important point is that the decay rate is equal to the constant γ without the factor $\sin\theta_{0}^{2}$ (when taking $\theta_{0}=\pi /2$ in these equations), and the non-diagonal components of the density matrix vanish at any time, as expected for an incoherent state.

This permits to understand where the $\sin^{2}\theta_{0}$ factor in the decay constant comes from. Let us associate this result with the Dirac expression for γ. In Dirac’s calculation, γ is proportional to the square modulus of the excited-state amplitude of the wave function because he considered a problem of evolution in general. From the point of view of Everett’s multiple worlds, this amplitude depends on the universe in which the atom evolves. If $\rho_{01}=0$, one knows that the atom may belong to the set of atoms that are in the excited state with a probability of one, and no reduction factor has to be associated with the decay rate γ. However, if $\rho_{01}\neq 0$, one cannot assume that the atom is in the excited state with a probability of one. Therefore, there is, a priori, a reduction factor (less than 1) to be included in Dirac’s formula for the rate γ.

## 3. Statistical Picture of the Emission of Photons and Everett’s Theory

**Figure 2:**
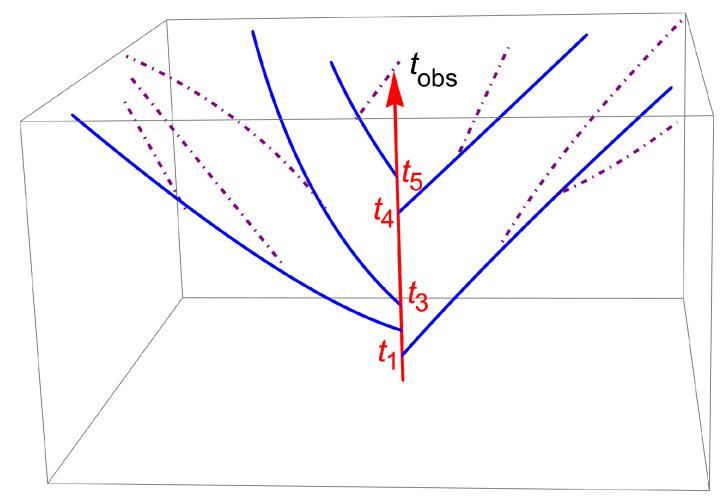
Schema of the possible trajectories of the atom emitting photons at times $t_{i}$, {i,1,5} in the universe of the observer. The vertical red line with the arrow is the trajectory seen by the observer, where the atom makes Rabi oscillations between $t_{i}$ and $t_{i+1}$. The solid blue lines stemming from each $t_{i}$ illustrate the successive splitting of the observer trajectory (universe) into two parts. On the blue trajectory (virtual for the observer), no photon is emitted at $t_{i}$, but Rabi oscillations go along until a photon is emitted in this universe. This occurs at the crossing points of the blue curves with the purple dotted–dashed curves. At these crossing points, a virtual “blue trajectory” splits into two parts, one (blue) with an emitted photon and another one (purple) with no photon emitted.

Let us return to the connection of our model with Everett’s theory that was presented in the 1950s for quantum physics, which is sometimes considered as philosophical speculation without a connection with real physics. As already mentioned in the introduction, Everett’s ideas are useful in understanding the statistical effects observed in fluorescence. One fundamental idea of Everett when applied to the problem of emission of photons by a single atom is that, after each the emission time $t_{i}$ the trajectory (or universe in Everett’s notation) of the system of an atom plus photons splits into two separate trajectories (or universes). One corresponds to the atom plus an emitted photon, which is the universe of the observer; the other one is the trajectory without an emitted photon, with the atom pursuing the Rabi cycles until a photon emission occurs in this universe. Each couple of universes $\left\{U_{i,1ph},U_{i,0ph}\right\}$ is indexed by the emitted photon {i}, which moves away from the atom at a given time $t_{i}$, so that the ensemble of all universes is nothing but an outflow of photons emitted at different instants. The important point is that all of these universes ignore each other, which implies no interference among them, a property justified because the characteristic time associated with a quantum jump is very short, of the order of the period of the atomic motion, which is also the period of the EM waves emitted by the atom in its excited state. This property allows us to make the Markov approximation leading to the Kolmogorov-like Equation (5) presented above and studied in [11]. A 3D schema illustrating a possible set of trajectories coming from successive $t_{i}$ is drawn in Figure 2 (see the captions) with the aim of illustrating that the various universes do not overlap.

By different universes, one implies two related things. First, the histories of the two universes are a priori different after the emission event. This does not imply a big difference, of course, between the two universes because their initial conditions at the instant of the emission are almost the same but for the absence or presence of a single photon. Secondly, the two universes are separated “mathematically” because their density matrices have no overlap. Therefore, one can define in each universe a density matrix that will evolve in the future without any relationship with the density matrix of the other universe. In the case of fluorescence, what happens in all universes can be described only statistically, the statistics being carried over all universes existing at a given time. This defines a kind of super-statistics because probability distributions are themselves defined over an object with a statistical meaning, namely, the density matrix for the quantum state in the universe under consideration. In the case of a pumped two-level atom, this density matrix depends on the angle θ so that the probability distribution is a probability depending on this single variable only.

Contrary to other theories of fluorescence of a single atom, such a statistical theory has a built-in statistical structure that is, we believe, necessary for describing the randomness of the emission process. Such a randomness is intrinsic to the emission process, represented as successive splitting of one trajectory into two every time a photon is emitted. By attempting to write a dynamical equation for the density matrix describing the emission process, one has to make a kind of average of this density matrix over all possible universes, something that is not physically possible because of the lack of overlap of the density matrices attached to the different universes.

## 4. Summary and Conclusions

The purpose of this paper was to show first how the view of quantum mechanics as a statistical theory grew from the very beginning of this theory and how things were clarified by Everett’s bold idea of multiple universes. We also felt that it was not sufficient to discuss these questions abstractly as points of metaphysics, but as those of physics (although the word “metaphysics” is not from Aristotle, it is here understood in its original meaning by Aristotle, as “just after physics”). This was demonstrated on a model problem with a non-trivial “solution”, namely, a model where the statistical analysis needs to be done very carefully even though its mathematics are actually fairly simple. This model also has the interest of being connected with the problems raised first by the founding fathers focused on the interaction of matter and light. We thought that it was instructive to show how the general concepts of quantum mechanics as a statistical theory work “concretely” in a given case. By “concretely”, we mean in a probabilistic mathematical framework using probability distributions and their evolution equation. We hope that this discussion of a specific model brings more light on this difficult subject than a more abstract discussion.

## Data Availability

Not applicable.

## References

[B1-entropy-23-01643] 1.We reluctantly use the word “equations” here because Newton never wrote down ordinary differential equations of classical mechanics in the modern sense. As is well known, he solved dynamical problems by using elegant geometrical methods instead of what we now call Calculus. For instance, the integral of the solution of the two-body problem with a general spherical potential was replaced by the calculation of areas between curves and two radii.

[2] Cornfeld I.P., Fomin S.V., Sinai Y.G. (1982). Ergodic Theory.

[3] Sinai Y.G. (1963). On the Foundation of the Ergodic Hypothesis for a Dynamical System of Statistical Mechanics. Sov. Math. Dokl..

[4] Heisenberg W. (1949). The Physical Principles of the Quantum Theory.

[5] Dirac P.A.M. (1927). The quantum theory of the emission and absorption of radiation. Proc. R. Soc. A.

[6] Everett H. (1957). Relative State Formulation of Quantum Mechanics. Rev. Mod. Phys..

[7] Einstein A. (1917). On the quantum theory of radiation. Phys. Z..

[8] Dehmelt H.G. (1975). Laser fluorescence spectroscopy on Ti+ mono-ion oscillator II. Bull. Am. Phys. Soc..

[9] Dehmelt H.G. (1982). Monoion oscillator as potential ultimate laser frequency standard. IEEE Trans. Instrum. Meas..

[10] Dehmelt H.G. (1987). Quantum jump. Nature.

[11] Pomeau Y., Le Berre M., Ginibre J. (2016). Ultimate statistical Physics: Fluorescence of a single atom. J. Stat. Mech..

[12] (1992). Probability theory and mathematical statistics. Selected Works of A.N. Kolmogorov.

[13] Lindblad G. (1976). On the generators of quantum dynamical semigroups. Comm. Math. Phys..

[B14-entropy-23-01643] 14.Starting from the Lindblad equation and using the dressed atom formalism, Reynaud, Dalibard and Cohen-Tanoudji reduce the set of infinite coupled equations (for coupled manifolds describing the fluorescent cascade) by the equations for a single manifold in their 1986 paper. Actually, this procedure amounts to truncating the Lindblad equation by suppressing the gain term, Γ*S*^−^*σS*^+^, in Equation (2). 1 of their paper. After truncation, the equation displays a nonconservative interaction Hamiltonian in the inter-emission intervals.

[15] Résibois P., de Leener M. (1976). Classical Kinetic Theory of Fluids.

[B16-entropy-23-01643] 16.We thank C. Cohen-Tannoudji, J. Dalibard, and S. Reynaud for a stimulating discussion that was at the origin of the above derivation.

[B17-entropy-23-01643] 17.We take the opportunity of this publication to give the right expression of the inter-emission time probability. In our 2016 paper with J.Ginibre, sin^2^($\frac{\Omega }{2}$*τ*) should be changed into sin^4^($\frac{\Omega }{2}$*τ*) in the expression of the conditional intensity of the photo-emission point process.

[18] Reynaud S., Dalibard J., Cohen-Tannoudji C. (1986). Photon statistics and quantum jumps: The picture of the dressed atom radiative cascade. IEEE J. Quant. Electr..

[19] Cohen-Tannoudji C., Dalibard J. (1986). Single-atom laser spectroscopy. Looking for dark periods in fluorescence light. Europhys. Lett..

[B20-entropy-23-01643] 20.In the 1986 paper of Cohen-Tannoudji and Dalibard, the authors interpret 1/*τ_Q_* as the width of the ground state induced by the pump laser. We must also notice that the average number of radiated photons per unit time deduced from the Bloch equations is also of the order *γ*/Ω^2^ in this limit.

